# Psychological assessment of parents of people diagnosed with borderline personality disorder and comparison with parents of people without psychological disorders

**DOI:** 10.3389/fpsyg.2022.1097959

**Published:** 2023-01-13

**Authors:** Verónica Guillén, Sara Bolo, Sara Fonseca-Baeza, Sandra Pérez, Joaquín García-Alandete, Cristina Botella, José Heliodoro Marco

**Affiliations:** ^1^Department of Personality, Evaluation and Psychological Treatments, Universidad de Valencia, Valencia, Spain; ^2^Ciber Fisiopatologia Obesidad y Nutricion (CB06/03), Instituto Salud Carlos III, Carlos, Spain; ^3^Department of Psicología Básica, Clínica y Psicobiología, Universidad Jaime I de Castellón, Plana, Spain

**Keywords:** evaluation, diagnosis, psychopathology, borderline personality disorder, relatives

## Abstract

**Background:**

To date, several evidence-based interventions have been created to help relatives of people with Borderline Personality Disorder (BPD), but few studies have analyzed the clinical situation of the family members. The aim of this study was twofold: (1) to explore the clinical symptomatology in a sample of parents of people diagnosed with BPD and compare them with a sample of a sample of people without a relative with a personality disorder, (2) to explore whether the parents of people diagnosed with BPD have psychopathology related to personality disorders (PD) or meet the diagnostic criteria for PD.

**Method:**

Participants were 42 (39.6%) fathers and 64 (60.4%) were mothers and mothers (*n* = XX, −%) of people diagnosed with BPD, who were selected from a specialized PD unit for treatment. The sample of people without a relative with a PD was obtained from social network announcements. To test for differences between the two groups, Student’s *t* tests were performed for quantitative variables, and Chi-square tests were performed for categorical variables. Cohen’s *d* was calculated as a measure of the effect size.

**Results:**

Parents of people with BPD showed greater depressive and anxious symptomatology, higher levels of expressed emotion, and worse quality of life than the sample of people without a relative with a personality disorder. In addition, a high percentage of the parents of people diagnosed with BPD (50%) met the diagnostic criteria for different PD.

**Conclusion:**

Parents of people diagnosed with BPD may need psychological help in various aspects. Therapists are therefore advised to bear in mind the importance of carrying out a psychological assessment of family members and, if necessary, to offer psychological intervention. It is crucial to invite the family to be part of the treatment, since they can be part of the solution.

**Clinical Trial registration**: ClinicalTrials.gov ID, NCT04160871 (registered November 15, 2019).

## Background

Borderline Personality Disorder (BPD) is one of the most frequent Personality Disorders (PD) in healthcare services, affecting between 1.2 and 6% of the general population (Grant et al., 2008) and up to 37% of the clinical population (Ryan et al., 2017). BPD is characterized by a “pattern of instability in interpersonal relationships, affectivity, and self-image, and significant impulsivity” (American Psychiatric Association, DSM-5 Task Force, 2013, p. 645), which begins in early adulthood and is generalized to different contexts. Likewise, people suffering BPD may present aggressive behaviors toward themselves and others, self-destructive behaviors and even suicide (Fernández, 2007).

One of the main characteristics of people with BPD is their style of relating to other people, which results in intense and unstable relationships, including their relatives. In fact, seven of the nine criteria for the BPD diagnosis have a direct effect on their relationships (Hoffman et al., 2007). Therefore, BPD symptoms affects not only the diagnosed person, but also their social environment (Giffin, 2008). The relatives of that people play a very important role in the evolution of the symptomatology of BPD.^42,43^ proposes that individual and environmental characteristics influence each other in triggering borderline symptomatology, and states that people with BPD start from a triple vulnerability: greater sensitivity to emotional stimuli, greater reactivity to emotional cues, and a slower return to their emotional baseline. This vulnerability and a lack of skills in the environment, that is, others’ responses to the patient’s internal experiences, contribute to the onset of emotional dysregulation (Fruzzetti and Iverson, 2006).

Given its strong individual and social impact, BPD has attracted the greatest interest among the scientific community in recent years (Zanarini M. et al., 2004). The treatment for BPD that has obtained the most empirical support is Dialectical-Behavioral Therapy (DBT; Linehan, 1993a b). However, despite the weight and responsibility that falls on family members, psychological treatments for BPD patients do not usually include their relatives (Glick and Loraas, 2001; Harman and Walso, 2001; Hoffman et al., 2005). In this regard, it is worth pointing out how difficult it is for relatives to live with BPD patients. In fact, they report feeling incapable of dealing with the problems of their loved ones. Giffin (2008) found that parents of daughters with BPD manifested chronic and traumatic stress, as well as feelings of guilt, social isolation, and exhaustion due to lack of sleep. Regalado et al. (2011) observed that 95.3% of family members present some degree of overload, as well as somatic symptoms, depression, anxiety, obsessions and compulsions, paranoid ideation, and other clinical symptoms like Scheirs and Bok (2007). These symptoms are greater in people whose relative with BPD has attempted suicide, and lack of appetite, sleep problems, guilt, and thoughts of death have also been observed. In this line, Goodman et al. (2011) found that 88% of relatives of patients with BPD perceived that their emotional health was severely affected, with this being the most impaired dimension, although they also showed problems related to their physical health, work, and interpersonal relationships. Other studies have found that family members of people diagnosed with BPD often experience severe forms of psychopathology (Gunderson and Lyoo, 1997). To this complex situation, the stigma that surrounds this disorder should be added. This stigma is observable not only in the general population, but also in some mental health professionals, who often refuse to care for this type of patient (Hoffman et al., 2005).

In a recent systematic review of interventions for BPD relatives, Guillén et al. (2021) describe the interventions created so far. Initially, some of these programs included the family in one or two sessions to give them guidelines for helping the patients (Blum et al., 2002; Rathus and Miller, 2002). Other programs included both patients and family members, with the treatment administered jointly (Santisteban et al., 2003, 2015), although the focus is on the BPD patient. Nevertheless, currently, there are programs where the treatment focuses exclusively on relatives of patients with BPD. Some of them present a psychoeducational format (Pearce et al., 2017; Grenyer et al., 2019), whereas others focus on skills training, either based on mentalization (Bateman and Fonagy, 2018) or on DBT skills (Hoffman et al., 2005, 2007). The program that has obtained the most empirical support so far is *Family Connections* (Hoffman et al., 2005, 2007; Flynn et al., 2017), which is based on DBT skills training and aims to create a validating family environment to deal with the constant ongoing crises (Liljedahl et al., 2019).

From our point of view, the fact that several specific programs have been developed for these families is a sign of progress. However, it is curious that, although evidence-based treatments for BPD relatives are already available, few studies have focused on obtaining information about the clinical and personal situation of family members of people with BPD. To date, the scientific literature on the topic suggests that relatives of people with a diagnosis of BPD show executive disfunctions (Gvirts et al., 2012) and response inhibition deficits (Ruocco et al., 2012), as a marker of heritability of BPD. Other studies have supported the heritability of cluster B personality disorders and specifically of BPD at about 0.70 (i.e., Coolidge et al., 2001; Torgersen et al., 2012), some of them highlighting the familial transmission of BPD features, finding that BPD features in mothers predict BPD symptoms in siblings longitudinally (i.e., Barnow et al., 2013).

In addition, literature focuses on the fact that relatives may lack the necessary skills to effectively help their loved ones (Fruzzetti et al., 2005; Hoffman et al., 2005, 2007; Wilks et al., 2016; Flynn et al., 2017). Other studies analyze the existence of clinical symptomatology in relatives of people with BPD and propose that this symptomatology may have contributed to the origin or maintenance of the BPD problem (Torgersen et al., 2000; Santisteban et al., 2003; Scheirs and Bok, 2007; Bailey and Grenyer, 2014; Ruocco et al., 2015).

The first proposal highlights parents’ difficulties and lack of skills in dealing with crises, emergency room visits, interpersonal conflicts, self-harm, or suicide attempts. Several studies point out that family members experience feelings of guilt, confusion, ignorance, incompetence (Buteau et al., 2008), depression, anxiety, and grief (Hoffman et al., 1999; Hoffman and Fruzzetti, 2007). This approach also emphasizes BPD patients’ difficulties in communicating and managing their emotions effectively, and the fact that, in conflict situations, the family environment manages to invalidate the person with BPD (Miller and Skerven, 2017). Consequently, a vicious circle often occurs where people who are invalidated in a generalized way do not learn emotional skills and often end up mislabeling their emotions, expressing them incorrectly, and invalidating themselves, while other people perceive them as chaotic, unpredictable, and emotionally intense, which leads to further invalidation (Fruzzetti et al., 2005).

The second proposal points out that the presence of psychological problems in family members may heighten the patient’s vulnerability. These studies show that genetic vulnerability and patients’ early negative experiences may increase the risk of developing BPD in adulthood (Steele et al., 2020). In this line, some studies analyze the stress associated with caring for people with severe mental illness (Baronet, 1999; Harvey et al., 2001; Veltman et al., 2002; Tsang et al., 2003; Östman and Hansson, 2004; Liu et al., 2007), finding that an environment with high emotional expression can worsen the patient’s psychopathology. In line with conflictive and unstructured family settings, Bandelow et al. (2005) showed not only a greater number of conflicts within the BPD family nucleus, but also a greater amount of mental illness among the parents, which would negatively influence the relationship and the maladaptive behaviors learned by their children. As far as we know, to date no studies have evaluated the different PDs in relatives of people with BPD or compared the clinical situation of BPD relatives and relatives of the normal population.

Therefore, the relationships and directionality among the different factors are not clear. However, given the importance of the family environment in the development and maintenance of BPD (Fruzzetti et al., 2005; Gunderson and Lyons-Ruth, 2008), we consider it relevant to examine this aspect more in depth. Thus, the aim of this study was twofold: (1) to analyze whether a sample of parents of people with BPD showed significant differences with a sample of people without a relative with a severe mental disorder on a number of relevant variables, such as depressive and anxious symptomatology, emotional expression, and quality of life; (2) to study whether there are differences in psychopathology related to personality disorders between the two samples and analyze whether PDs exist in the sample of relatives of patients with BPD.

## Methods

### Participants

Participants were 53 parents of people diagnosed with BPD (sample 1) and 53 parents of people without diagnosis of mental disorder (sample 2). Of the total number of participants, 64 (60.4%) were mothers and 42 (39.6%) were fathers. The ages of sample 1 ranged from 35 to 80 years (*M* = 54.63, *SD* = 8.19). Table 1 shows the sociodemographic characteristics of the both sample 1 and sample 2.

**Table 1:** Sociodemographic characteristics of the subsamples of relatives.

		Relatives of people with BPD (*n* = 47)		Relatives of general population (*n* = 47)	
		*n*	*%*	*n*	*%*
Parentage	Mother	32	60.4	32	60.4
	Father	21	39.6	21	39.6
Marital status
	Single	0	0.0	1	1.9
	Married	45	84.9	48	90.6
	Separated	6	11.3	3	5.7
	Widowed	2	3.8	1	1.9
Level of education
	Primary studies	23	43.4	14	26.4
	Secondary studies	15	28.3	16	30.2
	Higher education	15	28.3	23	43.4
Activity
	Student	0	0.0	4	7.5
	Employed	22	41.5	41	77.4
	Unemployed	31	58.5	8	15.1
Previous psychopathology
	Yes	9	17.0	3	5.9
	No	44	83.0	48	94.1
Current psychopathology
	Yes	10	18.9	2	4.0
	No	43	81.1	48	96.0
Age		*M* = 57.49 (*SD* = 8.10)		*M* = 51.77 (*SD* = 7.28)	

M, mean; SD, standard deviation.

Sample 1 was recruited from a group of parents of patients attending a specialized PD unit for treatment. They were offered participation in the study through the clinical personnel of the unit, and voluntarily accepted participation. The inclusion criteria were: (a) being a mother or father of a patient who met the criteria for BPD according to the Diagnostic and Statistical Manual of Mental Disorders 4th Edition (DSM-IV; American Psychiatric Association, 2000); (b) the patient had to have received a diagnosis of BPD by a clinical psychologist or psychiatrist; and (c) agreeing to voluntarily participate in the study by signing the informed consent document. The exclusion criterion was the presence of a severe psychopathology that made it impossible to carry out the evaluation (psychosis, schizophrenia, etc).

Sample 2 was obtained with the collaboration of third-and fourth-year psychology students, and were recruited through social networks announcements (mainly Facebook, WhatsApp, Twitter, Linkedin, and Instagram) using snowball sampling techniques, in which were sent a concise description of the project and request for participation. When relatives of the students accepted, only one of both parents was asked for participation. The inclusion criteria were: a) having children who had not received a diagnosis of BPD or any other clinical diagnosis by a psychologist or psychiatrist; and b) agreeing to participate voluntarily in the study by signing the informed consent form. The exclusion criteria were: a) having a child with a diagnosis of PD, BPD, or another mental disorder; and b) the presence of any severe individual pathology that made it impossible to carry out the evaluation.

The BPD patients (*n* = 28) were mainly women (*n* = 25, 89.3%) and had a mean age of 25.04 years (*SD* = 9.07). With regard to marital status, 75% (n = 21) were single, and 14.3% (*n* = 4) had a partner (Table 2). The patients were diagnosed with BPD by a clinical psychologist or psychiatrist after having the disorder for a mean of 7.88 (*SD* = 6.38) years, and the clinician rated the severity of the case, with a mean severity of 7.36 (*SD* = 1.85) out of 10. In addition, at the time of the study, these patients also presented comorbidity with different disorders such as Substance Abuse (27.3%), Anorexia Nervosa (20%), Major Depression (20%), Conversive Disorder (8%), Post-Traumatic Stress Disorder (4%), Unspecified Anxiety Disorder (4%), Obsessive–Compulsive Disorder (4%), Conduct Disorder (4%), and Attention Deficit Hyperactivity Disorder (4%).

**Table 2:** Sociodemographic characteristics of the patients.

		Patients (*n* = 28)	Total %
Gender	Woman	25	89.3
	Man	3	10.7
Marital status	Single	21	75.0
	Partner	4	14.3
Level of education	Primary studies	6	21.4
	Secondary studies	12	42.9
	Higher education	6	21.4
Dedication	Student	13	50.0
	Employed	2	7.7
	Unemployed	11	42.3
Severity	*M* = 7.36 (*SD* = 1.85)		
Years of development	*M* = 7.88 (*SD* = 6.38)		
Age	*M* = 25.04 (*SD* = 9.07)		

M, mean; SD, standard deviation.

### Measurement instruments

*Overall Anxiety Severity and Impairment Scale* (OASIS; Norman et al., 2006). We used the Spanish validation by Osma et al. (2019), a self-report instrument composed of five items rated from 1 to 4 for assessing the severity and impairment associated with different anxiety disorders, or subliminal symptoms when the criteria are not met. The Spanish validation used in this study confirmed the factor structure and obtained evidence of the scale’s good psychometric properties (Osma et al., 2019). In the present study, the OASIS showed an adequate internal consistency (α = **0**.92).

*Beck Depression Inventory-II* (BDI-II; Beck et al., 1996). We used the Spanish validation by Sanz et al. (2003), a self-report instrument that provides a measure of the presence and severity of depression from the age of 13 years. The Sanz et al.’s (2003) adaptation consists of 21 items related to sadness, anhedonia, pessimism, suicide attempts, and 15 other symptoms corresponding to DSM-IV and ICD-10 diagnostic criteria. The response options are Likert-type, and the items are scored on a four-point severity scale. The final score ranges from 0 to 69 points. This instrument has four cut-off points that differentiate between minimal depression, mild depression, moderate depression, and severe depression. In the present study, the BDI-II showed a high internal consistency (α = 0.95).

*General Self Efficacy Scale-12* (GSES-12; Sherer et al., 1982). We used the Spanish validation by Suárez et al. (2000), a scale that assesses personal competence to deal effectively with a variety of stressful situations, and it analyzes subjective beliefs about one’s abilities to handle certain situations. The GSES comprises 12 items that measure different aspects of personal competence, and it contains three subscales: initiative, effort, and persistence. It is a Likert-type scale with four response options (0 = It never happens to me; 4 = It always happens to me). The higher the score, the greater the sense of self-efficacy. The internal consistency estimate in the present study was α = 0.90.

*Level of Expressed Emotion Scale* (LEE; Cole and Kazarian, 1988). We used the Spanish validation by Sepúlveda et al. (2012), a self-report composed of 60 true-false items that measures the negative emotional climate within the family based on the caregivers’ perceptions of four aspects: attitude toward the disease, intrusiveness, hostility, and lack of tolerance or coping strategies. Scores range from 0 to 45 points, where higher scores indicate greater expressed emotion. In the present study, a Cronbach’s alpha of 0.92 was found.

*Quality of Life Index* (QOL; Mezzich et al., 2000). This scale is composed of 10 items with Likert-type scales ranging from 1 to 10 (1 = Poor; 10 = Excellent). It measures aspects related to physical, psychological, and emotional well-being, occupational and interpersonal functioning, socio-emotional, socio-community, and services support, personal and spiritual fulfillment, and the overall perception of quality of life. It is a dimensional scale where a higher score is related to better quality of life. In the present study, the internal consistency of the scale was adequate (α = **0**.93).

*Structured Clinical Interview for DSM-IV Axis II Personality Disorders* (SCID II; First et al., 1997). This instrument assesses the different personality disorders according to DSM-IV diagnostic criteria using a four-point Likert-type scale (1 = Absent; 2 = Subclinical; 3 = Present or true; 4 = Not enough information). The score can be used to formulate diagnoses both categorically and dimensionally. In addition, the interview includes a 119-item self-report with dichotomous responses that serves as a screening tool to expedite test administration. This tool also takes into account the passive-aggressive personality disorders and the depressive personality from Appendix B of the DSM-IV. In the present study, the internal consistency estimated with Cronbach’s alpha was adequate (α = **0**.998).

### Procedure

The total sample was non-randomized, convenience, and chosen due to accessibility and availability. On the one hand, sample 1 was collected through a specialized PD center in the city of Valencia, Spain. Their relatives were undergoing treatment at the clinical center and their parents were offered the chance to participate in the study. Once the study had been explained to them, they filled out the informed consent. Then, several clinical psychologists, experts in BPD at the clinical center, carried out the evaluation of the family members to verify that they met the inclusion and exclusion criteria. The evaluation lasted 1 h and consisted of a face-to-face clinical evaluation in which they were asked for information about their child’s problem, how they had managed it so far, and whether they had currently or previously received a clinical diagnosis. They were then given the evaluation protocol to complete at home. The assessment protocol consisted of a series of questions related to sociodemographic variables, followed by the clinical symptomatology assessment instruments (OASIS, BDI-II; GSES-12, LEE, QoL, and SCID-II). Among the questionnaires, the SCID-II self-report was included to analyze psychopathology associated to PD. When the parents of BPD patients scored positively on more than two traits on the SCID-II self-report, as indicated in the self-report indications, an expert PD clinician administered the SCID-II Structured Interview to confirm or rule out the diagnosis. Therefore, the parents who had positive scores on the SCID-II questionnaire were scheduled for a one-hour visit on another day for the SCID-II Structured Interview. For logistical reasons, this interview could not be carried out in the sample 2.

Sample 2 was obtained with the collaboration of the students from the Faculty of Psychology and Speech Therapy at the University of Valencia. Third-and fourth-year psychology students were asked for their voluntary collaboration in a study on family members. They were informed that the completion of the questionnaires would be anonymous and take about 45 min. The evaluation protocol was given to the students so that they could send it to their relatives. Once the parents had completed the protocols, the students handed them in. They were not offered any reward or grade increase for participating; they were simply encouraged to collaborate in the study.

All the participants in both sample 1 and sample 2 signed the informed consent describing the study objective and the voluntary nature of their participation. They were informed that all their data would be confidential and treated in accordance with the Organic Law of December 13th on Personal Data Protection (LOPD) and Royal Decree 1720/2007 of December 21st, which approved the Regulation on development of the Organic Law of December 13th on Personal Data Protection.

### Data analysis

The present study was descriptive non-experimental, whose purpose was to describe the characteristics of a sample of parents of people diagnosed with BPD and compare them to the characteristics of parents of people without diagnosis of mental disorder. Analyses were carried out using the statistical program SPSS for Windows, version 21 (IBM Corp, 2012). Descriptive statistics were calculated for the variables. To test for differences between the two groups, Student’s *t* tests were performed for quantitative and quasi-quantitative variables, and Chi-square (*χ*^2^) tests were performed for categorical variables. Cohen’s *d* was calculated as a measure of the effect size.

## Results

There were no differences between sample 1 and sample 2 in their marital status [*χ*^2^(3) = 2.430, *p* = 0.488], level of education [*χ*^2^(2) = 3.906, *p* = 0.142], or psychopathological [*χ*^2^(1) = 0.006, *p* = 0.939] and personal [*χ*^2^(1) = 3.137, *p* = 0.077] antecedents. However, differences were found in the age of the parents [*t* (104) = 3.820, *p* < 0.001], with the clinical sample being older, and the job situation [*χ*^2^(2) = 23.294, *p* < 0.001] with more unemployed people (*n* = 31) in the sample 1 than in the sample 2 (*n* = 8; Table 1).

When asked whether they currently suffer from any psychological problem, sample 1 showed higher rates of psychopathology [*χ*^2^(1) = 5.525, *p* = 0.019; *n* = 10] than the sample 2 (*n* = 2; Table 1). Sample 1 showed more psychological problems (Anorexia nervosa, *n* = 1; Major depression, *n* = 2; Bipolar disorder, *n* = 1; Anxiety disorder not specified, *n* = 3; Posttraumatic stress disorder, *n* = 1; Obsessive compulsive disorder, *n* = 1; Asperger’s disorder, *n* = 1) than sample 2 (Major depression, *n* = 3; Anxiety disorder not specified, *n* = 1; Table 3).

**Table 3:** Clinical diagnosis according to the relatives (DSM – IV).

	Relatives of people with BPD (*n* = 47)		Relatives of general population (*n* = 47)		Χ^2^
	*n*	%	*n*	%
Anorexia nervosa	1	1.9	0	0.0	1.033
Major depression	2	3.8	3	6.4	0.189
Bipolar disorder	1	1.9	0	0.0	1.056
Non-specified anxiety disorder	3	4.7	1	2.1	1.971
Post-traumatic stress disorder	1	1.9	0	0.0	1.033
Obsessive–compulsive disorder	1	1.9	0	0.0	1.033
Asperger’s	1	1.9	0	0.0	1.033

In general, sample 1 showed higher scores on several symptoms. There were statistically significant differences in the BDI-II scores measuring depressive symptomatology [*t*(104) = 2.904, *p* = 0.004, *d* = 0.56] and in anxious symptomatology measured with the OASIS (*t*(104) = 3.467, *p* = 0.001, *d* = 0.68; Table 4).

**Table 4:** Scores of relatives in the clinical population and relatives in the general population.

	Relatives of clinical population (*n* = 53) *M* (*SD*)	Relatives of General Population (*n* = 53) *M* (*SD*)	*t*	*d*
OASIS	4.75 (4.51)	2.28 (2.51)	3.467***	0.68
BDI – II	12.74 (10.96)	7.41 (7.61)	2.904**	0.56
Initiative (GSES – 12)	10.00 (1.92)	9.15 (2.33)	2.028*	0.40
Effort (GSES – 12)	13.92 (3.62)	12.32 (3.94)	2.163*	0.42
Persistence (GSES – 12)	12.51 (2.74)	12.71 (2.53)	−0.393	0.06
Total (GSES – 12)	36.43 (6.66)	34.19 (6.77)	1.710	0.33
Attitude toward the disease (LEE)	1.24 (2.58)	0.45 (0.79)	2.913**	0.41
Hostility (LEE)	2.68 (2.64)	1.59 (1.98)	2.366*	0.47
Lack of tolerance or coping strategies (LEE)	2.62 (2.50)	1.80 (2.41)	1.700	0.33
Intrusion (LEE)	2.68 (2.34)	2.13 (1.76)	1.328	0.27
Total (LEE)	9.23 (7.62)	5.10 (3.99)	3.400**	0.68
Physical well-being (QoL)	6.04 (2.05)	6.54 (2.10)	−1.237	0.24
Psychological well-being (QoL)	6.75 (2.13)	7.33 (1.98)	−1.426	0.28
Self-care (QoL)	8.13 (1.72)	8.50 (1.49)	−1.171	0.23
Occupational functioning (QoL)	8.09 (1.74)	8.69 (1.18)	−2.061*	0.40
Interpersonal functioning (QoL)	7.87 (1.85)	8.48 (1.42)	−1.901	0.37
Socio-emotional support (QoL)	7.42 (2.16)	8.58 (1.42)	−3.262**	0.63
Community support and from services (QoL)	7.57 (1.42)	7.62 (2.15)	−0.138	0.03
Personal fulfillment (QoL)	6.87 (1.99)	7.87 (1.79)	−2.696**	0.53
Spiritual fulfillment (QoL)	6.79 (2.18)	6.69 (2.54)	0.217	0.04
Overall perception of quality of life (QoL)	6.83 (1.84)	8.17 (1.49)	−4.108***	0.80
Total (QoL)	7.24 (1.46)	7.85 (1.35)	−2.225*	0.43

OASIS, overall anxiety severity and impairment scale; BDI-II, beck depression inventory-iI; GSES-12, general self-efficacy scale; LEE, level of expressed emotion scale; QoL, quality of life index. *p < 0.05; ** *p* < 0.01; ***p < 0.001.

In addition, statistically significant differences were found in self-efficacy, measured by the GSES - 12, on the Initiative [*t*(103) = 2.028, *p* = 0.045, *d* = 0.40] and Effort [*t*(103) = 2.163, *p* = 0.033, *d* = 0.42] subscales. The higher the score, the greater the sense of self-efficacy. In this case, sample 1 obtained higher scores than sample 2. However, no statistically significant differences were found on the Persistence subscale [*t*(98) = −0.596, *p* = 0.553, *d* = 0.12].

Regarding emotional expression measured with the LEE, statistically significant differences were found on the Negative Attitude toward the illness [*t*(74.691) = 2.913, *p* = 0.005, *d* = 0.41] and Hostility [*t*(96.623) = 2.366, *p* = 0.020, *d* = 0.47] subscales, with sample 1 obtaining higher scores than sample 2. There were no differences on the Intrusiveness [*t*(95.110) = 1.328, *p* = 0.187, *d* = 0.27] and Coping Strategies scales [*t*(101) = 1.700, *p* = 0.092, *d* = 0.33] subscales.

Finally, sample 1 obtained lower scores in quality of life than sample 2. That is, they perceived worse quality of life in several dimensions. Overall perception of quality of life [*t*(103) = −4.108, *p* < 0.001, *d* = 0.80], Socio-emotional support [*t*(90.018) = −3.250, *p* = 0.002, *d* = 0.63], Personal fulfillment [*t*(103) = −2.696, *p* = 0.008, *d* = 0.53], and Occupational functioning [*t*(103) = −2.061, *p* = 0.042, *d* = 0.40]. However, no significant differences were found on the Physical well-being [*t*(103) = −1.237, *p* = 0.219, *d* = 0.24], Psychological well-being [*t*(103) = −1.426, *p* = 0.157, *d* = 0.28], Self-care [*t*(103) = −1.171, *p* = 0.244, *d* = 0.23], Interpersonal functioning [*t*(103) = −1.901, *p* = 0.060, *d* = 0.37], Community support [*t*(88.138) = −0.139, *p* = 0.890, *d* = 0.03], and Spiritual fulfillment [*t*(103) = 0.217, *p* = 0.829, *d* = 0.04] subscales (Table 4).

With regard to the results obtained on the SCID-II Self-Report, statistically significant differences were observed in the obsessive–compulsive [χ2(1) = 8.134, *p* = 0.004] and antisocial [χ2(1) = 4.178, *p* = 0.041] personality dimensions between relatives of people with BPD and relatives of the general population, although these differences favored the normal population (Table 5). However, no differences were obtained in the avoidant [χ2(1) = 0.851, *p* = 0.356]; dependent [χ2(1) = 0.048, *p* = 0.826]; passive-aggressive [χ2(1) = 0.420, *p* = 0.517]; depressive [χ2(1) = 0.000, *p* = 1.000]; paranoid [χ2(1) = 0.696, *p* = 0.404]; schizotypal [χ2(1) = 0.737, *p* = 0.391]; schizoid [χ2(1) = 0.006, *p* = 0.937]; histrionic [χ2(1) = 0.641, *p* = 0.423]; narcissistic [χ2(1) = 2.785, *p* = 0.095]; or borderline (χ2(1) = 0.468, *p* = 0.494) personality dimensions (Table 5).

**Table 5:** Personality traits according to the SCID – II self-report in the two subsamples of relatives (DSM-IV).

	Relatives of people with BPD (*n* = 47)		Relatives of general population (*n* = 47)		χ^2^
	*n*	*%*	*n*	*%*
Avoidant	11	23.4	15	31.9	0.85
Dependent	15	31.9	16	34.0	0.048
Obsessive–compulsive	25	53.2	38	80.9	8.13*
Passive-aggressive	15	31.9	18	38.3	0.42
Depressive	16	34.0	16	34.0	0.00
Paranoid	18	38.3	22	46.8	0.69
Schizotypal	15	31.9	19	40.4	0.73
Schizoid	17	37.0	17	36.2	0.00
Histrionic	6	13.0	9	19.1	0.64
Narcissistic	16	34.0	24	51.1	2.78
Borderline	12	25.5	15	31.9	0.47
Antisocial	0	0.0	4	8.5	4.18*

*p < 0.05.

Table 6 shows the results of the SCID-II Structured Interview to confirm whether sample 1 showed a PD, compared to sample 2, according to the DSM-IV (American Psychiatric Association, DSM-5 Task Force, 2013). The data showed that 18.9% (*n* = 10) met the criteria for the diagnosis of Obsessive–Compulsive PD, 7.5% (*n* = 4) for the diagnosis of BPD, 5.7% (*n* = 3) for the diagnosis of Avoidant PD, 3.8% (*n* = 2) for the diagnosis of Histrionic PD, 3.8% (*n* = 2) for the diagnosis of Depressive PD, 1.9% (*n* = 1) for the diagnosis of Narcissistic PD, 1.9% (*n* = 1) for the diagnosis of Paranoid PD, and 1.9% (*n* = 1) for the diagnosis of Dependent PD. None of the participants met the criteria for the diagnosis of Passive-Aggressive, Schizotypal, Schizoid, or Antisocial PD. Therefore, 50% (*n* = 24) of sample 1 fulfilled the criteria for the complete diagnosis of a PD (Table 6).

**Table 6:** Personality disorder according to the SCID-II structured interview in the clinical population, compared to data from the DSM-IV (American Psychiatric Association, 2000).

	Relatives of people with BPD (*n* = 47)		Relatives of the general population according to DSM-IV (*n* = 47)
	*n*	*%*	*%*
Avoidant	11	23,4	2.4
Dependent	15	31,9	1.49–0.6
Obsessive–compulsive	25	53,2	2.1–7.9
Passive-aggressive	15	31,9	0
Depressive	16	34,0	0
Paranoid	18	38,3	2.3–4.4
Schizotypal	15	31,9	0.6–4.9
Schizoid	17	37,0	3.1–4.9
Histrionic	6	13,0	1.84
Narcissistic	16	34,0	0.0–6.2
Borderline	12	25,5	1.6–5.9
Antisocial	0	0,0	0.2–3.3

## Discussion

The purposes of this study were (1) to analyze whether there is clinical symptomatology in a sample of parents of people diagnosed with BPD (sample 1) and compare them to a sample of parents of people without a diagnosis of mental disorder (sample 2), and (2) to explore the personality of the parents of both samples in order to determine whether they had psychopathology related to PD or meet the diagnostic criteria for BPD.

The results indicated that sample 1 presented greater clinical symptomatology, specifically, higher levels of anxiety and depression than sample 2. The data showed that 22.6% of the sample 1 presented depressive symptomatology, and 11.3% presented anxious clinical symptomatology. These data are consistent with those found in previous research (Fruzzetti et al., 2005; Scheirs and Bok, 2007; Regalado et al., 2011) and support Carrotte’s thesis that, for every patient with a severe mental disorder, at least five relatives are directly affected (Carrotte and Blanchard, 2018). Other studies on relatives of patients with BPD found much higher levels of illness burden than in relatives of patients with other severe mental disorders, especially if the patient is young, presents self-injury behaviors, or engages in suicide attempts (Bailey and Grenyer, 2014; Osma et al., 2019).

Regarding the sense of self-efficacy, our results indicated that sample 1 obtained statistically significant higher scores on relevant variables such as Initiative and Effort than sample 2. According to these data, the parents of people diagnosed with BPD perceived themselves as having higher levels of initiative and effort in managing the problem and the relationship with their children. These findings are similar to those obtained in previous studies (Guillén et al., 2018), which show that the parents of people diagnosed with BPD have to make a greater effort and take the initiative to offer help to their children with BPD more often in order to adapt to their needs, compared to family members of non-clinical population. However, no differences were found on the persistence variable between both samples. Caring for the children might involve persistence in both populations.

With regard to expressed emotion, statistically significant higher scores were also found in sample 1 than in sample 2. Specifically, they more frequently showed a negative attitude toward their children’s illness and greater hostility toward the patients and the family situation in general. Likewise, fewer coping skills were also observed in the sample 1, and though the differences in this dimension were not statistically significant, they had a large effect size. Thus, it is possible that a larger sample size would have yielded statistically significant differences. In general terms, these results indicate that sample 1 found it more difficult to accept the illness and manage hostility. Other studies in this line indicate that high levels of expressed emotion are related to greater psychological distress in caregivers (Kyriacou et al., 2008; Sepúlveda et al., 2012; Sadiq and Suhail, 2019).

Moreover, sample 1 perceived their quality of life as less satisfactory than sample 2 on relevant variables such as occupational functioning, socio-emotional support, personal or spiritual fulfillment, the overall perception of quality of life, and the total perception. Therefore, sample 1 showed worse quality of life than sample 2. These results coincide with those found in other studies with caregivers (Hayes et al., 2015; Farina et al., 2017; Zhang et al., 2018). In this regard, the high unemployment rate presented by sample 1 (58.5%), compared to sample 2 (15.1%), stands out. Therefore, taking into account the high psychosocial impact of caring for a dependent person, it is possible that this high rate is due to the fact that family members devote much of their time to caring for their loved one. This dedication would affect their occupational functioning, as well as their personal fulfillment and perception of the socio-emotional support available to them.

With regard to the second objective of this study, that is, to explore whether psychopathology related to PD (as measured with the SCID-II Self-Report) exist in both samples, the results indicated that sample 1 did not differ from sample 2, except on obsessive–compulsive PD and antisocial PD, which were higher in sample 2. However, as the SCID-II self-report indicated, these results should be viewed with caution because the use of the SCID-II Interview is required in order to confirm or rule out a PD. In fact, when clinical psychologists administered the SCID-II Structured Interview to parents of people diagnosed with BPD, the results indicated that in sample 1 many people met the criteria for various PDs. Thus, almost half of sample 2 (24 out of 53 BPD family members) met the diagnostic criteria for a PD. On a disorder-by-disorder basis, Obsessive–Compulsive PD (18.9) was the most frequent, followed by BPD (7.5%), Avoidant PD (5.7%), Histrionic Personality Disorder (3.8%), Depressive PD (3.8%), and, less frequently, Narcissistic PD (1.9%), Dependent PD (1.9%), and Paranoid PD (1.9%). As noted above, to date, no studies have evaluated the different PDs in relatives of people with BPD or compared the clinical situation of BPD relatives and relatives of the normal population. In this regard, it is important to note that people who meet the criteria for PD show difficulties adapting to others and the environment. In addition, people with PD show a pattern of inflexible and generalized rigidity that significantly hinders their daily functioning, as well as the performance and achievement of tasks or relationships with others. The impact of this problem is usually reflected in the different areas of their lives (e.g., cognition, affectivity, interpersonal areas, and impulse control), and it remains stable over time, so that a high percentage of people who suffer from it develop comorbidity with other pathologies throughout their lives. In addition, it usually produces clinically significant discomfort and interference (American Psychiatric Association, DSM-5 Task Force, 2013. The study by Zanarini M.C. et al. (2004) proposes that relatives of people with BPD may present the typical symptomology of this disorder (inappropriate anger, impulsivity, emotional instability, dissociation, and intense interpersonal relationships) to a greater extent than family members of people with other PD. Likewise, other studies show that the role of the parents’ PD, in this case, the mother’s BPD, has a noteworthy influence on the formation of attachment in her offspring, who show higher rates of insecure attachment or disorganized attachment compared to healthy controls (Choi-Kain et al., 2009). Other studies, such as the one by Reinelt et al. (2013), indicate that mothers with BPD who alternate between two parenting styles, overprotection and rejection, may influence in the development of BPD features, making it difficult for the children to predict their mother’s behavior. Mahan et al. (2018) found a direct relationship between overprotection and the invalidating environments that these mothers form around their daughters when they reach adolescence and the subsequent development of the disorder in these girls. However, no studies have been found that relate them to obsessive–compulsive PD or other PD in the parents.

Therefore, these results indicate that the relatives of people with BPD have more psychological problems than the community sample. On the one hand, they show greater clinical anxiety, depression, problems with emotional expression, and functioning difficulties in different areas of their lives. In addition, the relatives of the clinical sample fulfill criteria for more personality disorders than the non-clinical sample of relatives. Therefore, their clinical symptomatology may have an influence on the relative with a BPD diagnosis, or in the family environment. Hence, it is possible that the relatives’ symptomatology might be influencing the maintenance of the borderline symptomatology in their children. It would be necessary to clarify to what extent this clinical symptomatology might have been present previously, and whether or not it could have influenced the origin of the disorder, as other authors have found (Barnow et al., 2013). Undoubtedly, people who experience higher levels of clinical symptoms or a PD may have more personal difficulties, which could lead to problems in their relationships with others, in the parenting system, or in their relationships with their family. However, with these data, it is not possible to determine to what extent the clinical problems of the relatives were already present and contributed to the genesis of BPD. Therefore, it would be necessary to carry out additional studies using longitudinal designs to obtain information about the clinical situation of the relatives of people with BPD at various times in their lives, as well as the situation of their children, in order to establish a causal relationship.

### Limitations and suggestions for further research

The results of the present study should be considered within the limitations of this research. First, there are limitations related to the sample size (*n* = 106). It would have been desirable to obtain a larger sample because a small sample can affect the power of the analyses. Moreover, it should be noted that the groups were not homogeneous on all the demographic variables, age, and employment status, given that the clinical sample was somewhat older and more of them were unemployed. Furthermore, due to the cross-sectional design, it is not possible to establish a causal association between being the parent of a child with BPD and presenting psychopathology, which could be a consequence of the disorder or exist prior to parenthood. Also, it would have been desirable to use the Structured Clinical Interview for DSM-5 (SCID-5) version, but we used the interview version that clinicians were using with their patients at the time. In addition, it would have been desirable to evaluate the non-clinical sample with the SCID-II Structured Interview, but due to time and space constraints, it was not possible. Finally, using the parents of psychology students as part of the community sample could bias the results because students sometimes choose this discipline in order to find an explanation for their experiences or an answer to certain problems.

## Conclusion

The results of this study make a modest contribution to the field of family members. It is surprising that there are as many as 11 empirically supported intervention programs for relatives of people with BPD (Guillén et al., 2021), but there are almost no evaluation studies focused on relatives. However, from our point of view, what is relevant in our clinical practice as psychologists or psychiatrists is to systematically analyze the clinical situation of family members in order to determine whether they need psychological help and what type of help would be most effective. Second, it is important to try to offer help to all those who need it. These data show that parents of people diagnosed with BPD have more clinical symptomatology and traits or PD than parents of people without a diagnosis of mental disorder, and so they require psychological help that they usually do not find in their environment. Clinicians tend to take care of the patient due to the seriousness and urgency involved, and they leave the relatives aside. Once again, what is important takes second place to what is urgent.

## Data availability statement

The original contributions presented in the study are included in the article/supplementary material, further inquiries can be directed to the corresponding author.

## Ethics statement

The studies involving human participants were reviewed and approved by The Ethics Committee of Research in Humans of the Ethics Commission in Experimental Research of University of Valencia (INV_ETICA_1955599). The patients/participants provided their written informed consent to participate in this study. Written informed consent was obtained from the individual(s) for the publication of any potentially identifiable images or data included in this article.

## Author contributions

VG drafted the manuscript with important contributions from JG-A and CB. VG and JG-A designed the study and participated in each of its phases in collaboration with JM. SB, SF-B, and SP carried out the assessments of family members. All the authors participated in the review and revision of the manuscript and approved the final manuscript to be published.

## Funding

Funding for the study was provided by grants: Regional Ministry of Innovation, Universities, Science and Digital Society: Subsidies for Consolidable Research Groups, − AICO/2021 (Generalitat Valenciana) has been awarded to VG as the main investigator of the project.

## Conflict of interest

The authors declare that the research was conducted in the absence of any commercial or financial relationships that could be construed as a potential conflict of interest.

## Publisher’s note

All claims expressed in this article are solely those of the authors and do not necessarily represent those of their affiliated organizations, or those of the publisher, the editors and the reviewers. Any product that may be evaluated in this article, or claim that may be made by its manufacturer, is not guaranteed or endorsed by the publisher.

## Abbreviations

BPD: borderline personality disorder
PD: personality disorder
DBT: dialectical-behavioral therapy
FC: family connections
DSM-IV: diagnostic and statistical manual of mental disorders, 4th edition
SD: standard deviation
OASIS: overall anxiety severity and impairment scale
BDI-II: the beck depression inventory-ii
GSES: general self-efficacy scale-12
LEE: level of expressed emotion scale
QOL: quality of life index
SCID II: structured clinical interview for DSM-IV axis II personality disorders
LOPD: organic law of December 13th on personal data protection
